# Polybrominated Diphenyl Ether (PBDE) Serum Concentrations in Italian Women of Reproductive Age

**DOI:** 10.3390/toxics14010072

**Published:** 2026-01-13

**Authors:** Annalisa Abballe, Elena De Felip, Elena Dellatte, Nicola Iacovella, Valentina Marra, Roberto Miniero, Silvia Valentini, Anna Maria Ingelido

**Affiliations:** Unit of Human Exposure to Environmental Contaminants, Department of Environment and Health, Istituto Superiore di Sanità, 00161 Rome, Italy; annalisa.abballe@iss.it (A.A.); annamaria.ingelido@iss.it (A.M.I.)

**Keywords:** women of reproductive age, PBDEs, human exposure, Italy

## Abstract

The evaluation of human exposure to environmental contaminants is a highly relevant topic for carrying out appropriate risk assessments and management. For this reason, although exposure assessment studies are continuously increasing, it is important to increase knowledge on the subject, especially when data gaps exist. Polybrominated diphenyl ethers (PBDEs) are a class of substances for which the available data in the literature are not abundant compared to other more studied contaminants. In particular, the data available for the Italian population are even more limited. This study aimed to characterize the exposure of women of reproductive age to PBDEs in different Italian regions. We focused on the study on women of reproductive age because they are a sensitive category, and, furthermore, the exposure of mothers allows us to estimate that of newborns. Study results showed that the most abundant congeners in terms of relative concentration were BDE-153 > BDE-47 > BDE-100 > BDE-99, with median estimates, respectively, of 0.670, 0.245, 0.110, and 0.100 ng/g lipid in serum samples. Overall, the average exposure of the study population to the selected flame retardants appears to be relatively low compared to other industrialized countries. The observed levels could be related to the decline of PBDE concentrations in Europe due to a ban in the European Union on most PBDE commercial technical mixtures from 2001 onwards.

## 1. Introduction

Polybrominated diphenyl ethers (PBDEs) are a class of synthetic flame-retardant chemicals with a basic structure consisting of two phenyl rings linked by an oxygen atom. PBDEs are commonly used to reduce flammability of many different materials [1]. As they are mixed into polymers and not chemically bound to plastic or textiles, they might be separated or leach from products into the environment [2] through mechanisms such as abrasion, combustion, and uncontrolled disposal of waste [3]. PBDEs have been widely added to items like foam cushioning and fabrics in sofas/chairs and plastics for electrical and electronic equipment [4,5,6].

Because of their high chemical–physical stability and long environmental and biological persistence, these lipophilic compounds can accumulate in organisms and biomagnify [3,7,8]. Thus, they have become ubiquitous in the environment and in the human population [9,10,11]. PBDEs have been detected in air, sediments, surface water, fish, and other marine animals [12,13,14,15]. Lower brominated congeners of PBDE bioaccumulate more than higher brominated congeners and are more persistent in the environment [12,16].

Studies on animals and humans have shown that some PBDEs can act as endocrine system disruptors and tend to deposit in human adipose tissue and breast milk [7,10,12,17,18]. Several studies have shown that overexposure to PBDEs causes liver injuries, thyroid hormone disorder, neurotoxicity, genotoxicity, and immune system disorders [7,18,19,20].

In 2008, the United States Environmental Protection Agency (US-EPA) issued health assessments of four individual PBDE congeners, BDE-47, -99, -153, and -209, within its Integrated Risk Information System (IRIS) program. These four congeners were those most found in the environment and in humans, and for which toxicological studies suitable for dose response assessments were available [21]. The same ones were also taken into consideration by the European Food Safety Authority [8,22] to develop advice for human consumption. The International Agency for Research on Cancer (IARC) classified PBDEs as “probably carcinogenic to humans” (Group 2A) based on mechanistic similarities to polychlorinated biphenyls [1].

The three commercial technical mixtures of PBDEs are PentaBDE, OctaBDE, and DecaBDE. They are composed of a mixture of congeners and are named according to their average bromine content [2]. Since August 2004, the use and import of products containing more than 0.1% penta- or octabromodiphenyl ethers have been banned in the European Union [23]. Consequently, the deca-mixture (containing around 97% of decaBDE) is more frequently than ever the main PBDE mixture in use nowadays [24]. In May 2009, tetrabromodiphenyl ether BDE-47, pentabromodiphenyl ether BDE-99, hexabromodiphenyl ethers BDE-153 and BDE-154, and heptabromodiphenyl ethers BDE-175 and BDE-183 were added to Annex A (Elimination) of the Stockholm Convention on Persistent Organic Pollutants (POPs) as they meet the criteria for so-called persistent organic pollutants of persistence, bioaccumulation and toxicity [25]. Decabromodiphenyl ether (PBDE-209) was added to Annex A of the Stockholm Convention in May 2017 through Decision SC-8/10 [26].

In recent decades, many human biomonitoring studies have been carried out: PBDE levels were measured in blood/serum/milk samples in North America, Europe, and Asia. The National Health and Nutrition Examination Survey (NHANES) and the German Environmental Survey (GERES) are two examples of public health studies assessing population exposure to environmental pollutants (including PBDEs) via human biomonitoring in the USA and Germany, respectively. No national human biomonitoring program exists in Italy, and a few human exposure data on PBDE are available. PBDEs were included in the human biomonitoring project “Womenbiopop” (April 2010–May 2013). This project was aimed at characterizing the exposure of women of reproductive age to a group of priority environmental contaminants and exploring a possible correlation between exposure and women’s reproductive health. Indeed, PBDEs have been associated with adverse effects on reproductive functions and adverse effects on male and female reproductive health and on neonatal health. For these reasons, it is very relevant to gather information about the exposure level of women of reproductive age. Articles reporting results about women’s exposure to dioxins, furans, polychlorinated biphenyls, pesticides, and PFAS have already been published [27,28,29]. The concentrations of these analytes found in women were comparable with those in other countries, with most falling in the lower end of the range. Geographical differences in concentrations were observed for almost all analytes. Overall, although a decline over time was observed for almost all analytes, the significant differences between geographic areas highlighted that significant exposure differences still existed for these analytes. This paper focuses on concentrations of PBDEs; it will contribute to expanding knowledge on female exposure to flame retardants in Italy. The results may also contribute to expanding knowledge about PBDE exposure in Europe.

## 2. Materials and Methods

### 2.1. Recruitment of Women and Questionnaires

Study participants were enrolled in six Italian regions. In northern Italy we enrolled participants residing in Trentino-Alto Adige and Piemonte; in central Italy, in Umbria and Lazio; and in southern Italy, in Puglia and Sicilia. Industrial, rural, and urban areas were included in sampling locations. The study design is reported elsewhere [28,29]. We enrolled about 500 nulliparous women aged between 20 and 40 years. The study was approved by the Ethics Committee. The participants donated blood, signed an informed consent form, and answered a detailed questionnaire related to anthropometric and socio-demographic characteristics and lifestyle factors.

### 2.2. PBDE Analysis

Serum samples (approximately 25 mL each) were added with 50 μL of 13C—labeled internal standards (Cambridge Isotope Laboratories, Inc., Tewksbury, MA, USA; concentration of 129 ng/mL for BDE-28, BDE-47, BDE-99, BDE-100, BDE-153, BDE-154, and BDE-189; 1290 ng/mL for BDE-209. Spiked samples were given of 10 mL formic acid and iso-propanol 1:1 (CARLO ERBA Reagents srl, Cornaredo (Milano), Italy) and sonicated for 10 min. The procedure was repeated two times. Subsequently, samples were extracted with 8 mL of n-hexane (CARLO ERBA Reagents srl, Cornaredo (Milano), Italy) and centrifugated at 1900 rpm for 10 minutes (Centrifuge Megafuge 1.0R, Heraeus Sepatech, Hanau, Germany). The extraction was repeated two times. N-hexane extracts were concentrated under a nitrogen stream and subjected to lipid removal by elution on a glass column containing Extrelut™ (VWR International, Milan, Italy) impregnated with concentrated sulfuric acid (CARLO ERBA Reagents srl, Cornaredo (Milano), Italy). The pre-purified extracts were cleaned up with an automatic Power—Prep™ system (FMS—Fluid Management Systems, Inc., Watertown, MA, USA) [30]. All the PBDE concentrations were adjusted to the lipid contents of the serum. Total cholesterol and triglycerides were determined by enzymatic methods by the Laboratory of Clinical Pathology, Università di Roma “Sapienza”, and total lipid content was calculated by Rylander’s formula [28,31].

A total of eight PBDE congeners were measured, BDE-28, BDE-47, BDE-99, BDE-100, BDE-153, BDE-154, BDE-189, and BDE-209, but only the BDE-47, BDE-99, BDE-100, and BDE-153 congeners were found to be above the limit of quantification (LOQ) in 37–95% of study participants and reported in this paper. PBDEs were quantified by isotopic dilution using an HRGC-HRMS (Thermo-DFS, Thermo Fisher Scientific Inc., Waltham, MA, USA), applying the selected ion monitoring mode (SIM). The instrument was equipped with a J&W (Agilent, Santa Clara, CA, USA) VF-1MS fused silica capillary column (15 m long, 0.15 mm internal diameter, and 0.15 µm film thickness). Detailed instrumental parameters are reported in the Supplementary Materials. In each batch we inserted a laboratory reference material and a blank sample. Blank samples consisted of hexane (CARLO ERBA Reagents srl, Cornaredo (Milano), Italy) in the same volume as the serum samples to be analyzed. As we did not have any certified reference materials available, we used a homemade pool of serum samples as a reference material (RM). The pool had previously been analyzed for the construction of the control chart. In each batch, we had one blank and one portion of the RM in a volume similar to the sample to be analyzed. A medium bound approach was applied. The limits of quantification were in the range of 0.01–2 ng/g lipid, depending on the PBDE congener. Recovery rates were in the 35–115% range [32].

### 2.3. Statistical Approach

We administered, to the participants, a targeted questionnaire to identify potential factors influencing the body burden of pollutants. Personal, environmental, and dietary variables included in the questionnaire were initially checked for missing values, and their relative frequencies were estimated. The descriptive statistical approach includes the definition of the distribution frequency type, which was estimated by the Maximum Likelihood Estimation (MLE) approach and parameters describing it (Statgraphics Centurion XVIII, Statpoint, Herndon, VA, USA). The above-mentioned evaluation was carried out on the observed values including the LOQ/2 data and excluding the missing data. We chose this approach because the high missing rates (Table S1) could influence the study of the relationships between the chemical concentrations and the covariates. Subsequently, the selected variables were analyzed using a multiple linear robust regression approach (Stata 16, Stata Corp, College Station, TX, USA). We used the Variance Inflation Factor (VIF) to assess collinearity among independent variables. We developed the final models through an iterative process: starting with a regression model including all significant variables, variables were progressively eliminated based on their *p*-values until the model achieved maximum statistical reliability. A *p*-value threshold of *p* ≥ Maldonado and Greenland [33]. We conducted further statistical analyses, including marginal means and marginal predicted choice probabilities, and adjusted predictions based on the previously fitted models. These calculations were performed at fixed values of the covariates and averaged over the covariates of interest. This post-estimation strategy provides estimates that facilitate the interpretation of the results from a choice model (Stata 16).

## 3. Results and Discussion

**Figure 1:**
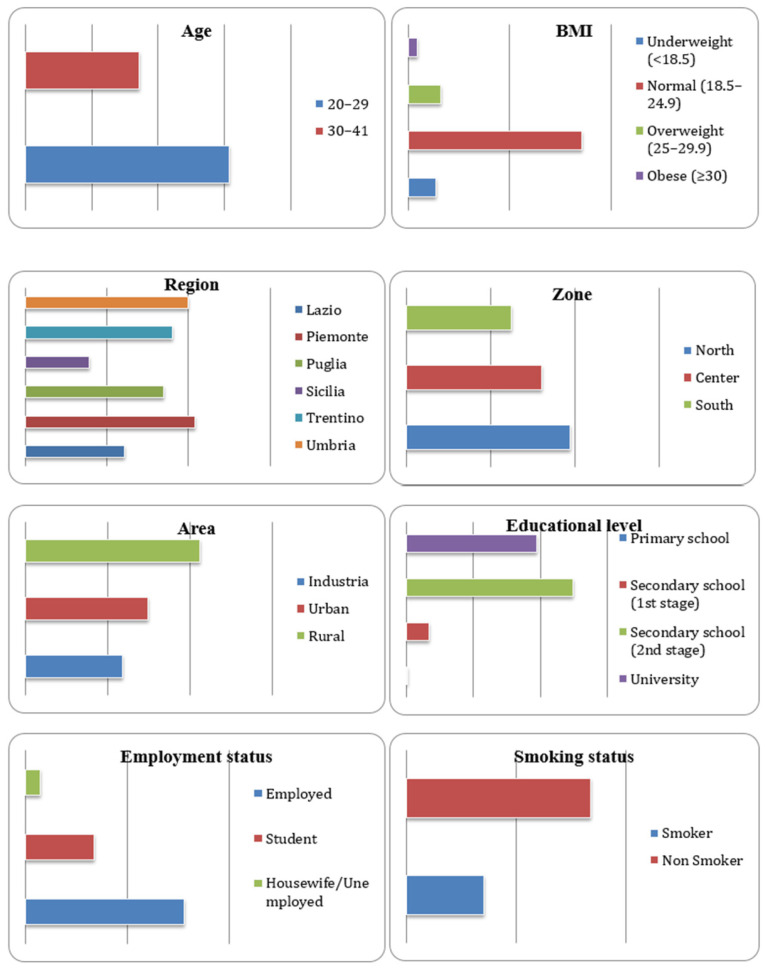
Anthropometric and socio-demographic characteristics of study participants.

Characteristics of study participants such as age, body mass index (BMI), region, geographical zone, typology of residence area, educational level, employment status, and smoking habits are shown in Figure 1. The mean age of participants was 27.7 years. Most subjects (64%) were in the age class 20–29 because of difficulties in finding women who had never breastfed in the age class >30 years. Most women (71%) showed a normal BMI; the mean value was 22.2 kg/m^2^. Forty-one percent of study participants were from northern Italy, 34% from the Center, and 26% from the South. A total of 44% were from rural areas, 31% from urban areas, and 25% from areas with industrial settlements. A total of 93% of the women enrolled had a medium to high educational level, and 65% were employed. About 30% of participants were smokers. The women from Trentino Alto Adige were significantly younger (Kruskal–Wallis, *p* << 0.01) than the women from the other regions.

The characterization of the BDE-47, -99, -100, and -153 data, according to the log likelihood statistic, showed that the best-fitting distribution was the log normal distribution [34]. A log-likelihood statistic measures how well a statistical model fits observed data by calculating the natural logarithm of the likelihood function, transforming products of probabilities into more numerically stable sums, making it ideal for parameter estimation and model comparison. The detected concentrations of each congener showed a high rate of missing values (Table 1) and also included the contribution of LOQ/2 values.

**Figure 2:**
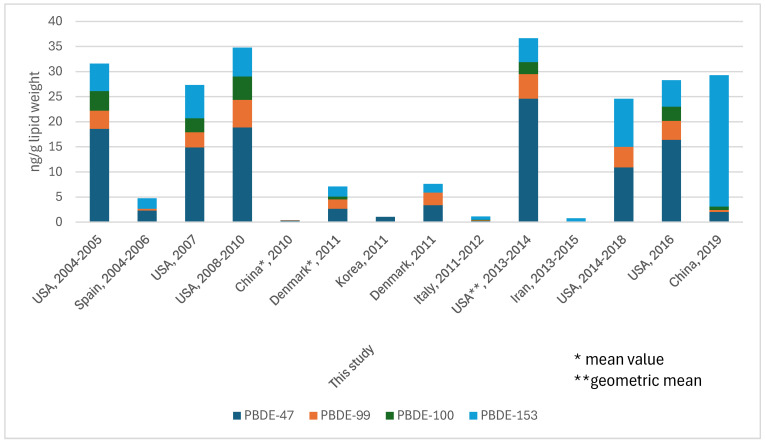
Median serum PBDE concentrations (ng/g lipid) in women from different countries in the period 2004–2019.

In this table the most frequently detected congeners were BDE-153 > BDE-100 > BDE–99 > BDE–47 (range 467–318) but, considering their median values, the most abundant in terms of relative concentration pattern to the total quantity (Σ_4_PBDE) were BDE–153 > BDE–47 > BDE–99 > BDE–100. The PBDE profiles found in the serum of the women in the study were consistent with those found in studies on PBDEs in food and house dust in Italy [35,36,37]. The PBDEs detected in higher concentrations in the serum of the women in the study are, in fact, always present among those measured at relatively high concentrations in food and house dust. Considering the potential for bioaccumulation in humans, the profiles of these four substances were consistent in humans and in environmental matrices. Furthermore, PBDE concentrations detected in food in Italy were comparable to those in other countries [35,37], and the authors [35] concluded that the exposure levels detected should not pose any particular risk to human health. This finding is consistent with the internal doses found in this study. In Table 1 the descriptive statistical evaluations were reported, and the descriptors X_MIN_, P_25_ (P = percentile), P_50_, geometric mean, arithmetic mean, P_95_, P_75_, X_MAX_, and N, used by default, are shown. BDE-47, -99, -100, and -153 concentrations fall in the ranges 0.01–42.0, 0.01–156.7, 0.01–7.20, and 0.04–3.38 ng/g lipid, respectively; the related median and geometric mean estimates are 0.245 and 0.169, 0.100 and 0.111, 0.110 and 0.112, and 0.670 and 0.654 ng/g lipid. These congeners appear to be among the most frequently detected in human serum studies [38,39,40,41], as shown in Figure 2.

In Figure 2 central tendency descriptors of the concentrations of the above-mentioned congeners determined in women groups with overlapping age ranges from different studies carried out in the years 2004–2019 are reported. Across the years 2004–2012 in Spain, Denmark, and Italy, exposure to the four congeners apparently decreased ([38,41,42]; this study); this is possibly due to the 2004 strict ban put on the use of commercial penta- and octa-BDE mixtures in the EU.

In the USA, although prohibitory and replacement actions against PBDEs started to appear [43], exposure to them in the years 2004–2014 did not decrease. However, a recent decrease was also observed in the USA, corresponding probably to a change in the profile due to the effects of the above-mentioned prohibitory and replacement actions because the congener BDE–100, a penta-substituted substance, was not determined. However, in the USA, the levels remain much higher than in other countries. In parallel, in China, in the years 2010–2019, exposure to the four congeners increased many times over, also showing a deep change in the exposure profile which was recently dominated by the BDE–153. On the contrary, BDE–47 appeared to be the predominant congener in serum samples until 2018, apparently subsequently substituted by BDE–153. The BDE–99 and –100 concentrations seem to be comparable in American studies until 2010, while in Spain, Denmark, and Italy, exposure to BDE–99 exceeds exposure to BDE–100 [39,40,44,45,46,47]. For all the congeners taken into consideration, the data distributions are asymmetrical and right—skewed. In Table 2, all the participants’ resultant outliers in the data distributions were calculated by modified Z-score. One participant (indicated with the ID 281 in Table 3), who lives very high concentrations of BDE-47, BDE-99, and BDE-153. At the same time, one woman living in the Piedmont Region (indicated with the ID 384 in Table 3) shows very high concentrations of BDE-47 and BDE-99. However, four out of six women in Table 3 live in the Umbria region.

We compared the above-mentioned data on the outliers with possible health guidance values such as Biomonitoring Equivalents (BEs) [48]. This approach identifies the concentration of a chemical in a biological medium such as blood, which is consistent with defined exposure guidance values or toxicity criteria, including reference doses and reference concentrations, minimal risk levels, or tolerable daily intakes [48]. Until now, only a BE for the BDE-99 has been developed, which can be compared with a participant from the Umbria Region who showed the highest concentration (156 ng/g lipid). This concentration was approximately lower than 1/3 of the corresponding bio-equivalent concentration (520 ng/g lipid) [49]. However, the same participant also showed a high concentration of BDE-47. The presence of relatively high concentrations of PBDEs in individual study participants and in population subgroups indicates that, despite the strong reduction in these contaminants in the environment and in humans, it is still useful to continue monitoring their concentrations.

In Table 2 and Table 4, the results of the robust regression approach are reported. In both the tables, only BDE-99 and BDE-100 showed significant results (*p* ≤ 0.05), possibly because the potential sources are still active. Both the chemicals were added to Annex A (Elimination) of the Stockholm Convention in May 2009, as well as BDE-47 and BDE-153, but, evidently, unlike previous PBDEs, their elimination was less effective. Table 2 displays the adjusted means and 95% confidence intervals for the categories of BMI, while Table 4 displays the concentrations of BDE-99 and BDE-100 marginal means and 95% confidence intervals for the regions. BDE-99 concentration showed a marginal increase in direct proportion to BMI across the interval from 18.5 to 29.8. Following this range, the concentration did not increase in the next interval (30.1–37.2), showing no statistically significant differences from the baseline value (Table 2). On average, the concentrations of BDE-99 and BDE-100 were of a comparable order of magnitude when comparing the same regions (Table 4). The concentration range between them was 0.084–0.147 and 0.083–0.131 ng/g lb, respectively. The most significant differences, however, were observed between regions: Latium exhibited the lowest PBDE average concentrations (BDE-99, 0.084 ng/g lb; BDE-100, 0.083 ng/g), while Sicily showed the highest (BDE-99, 0.147 ng/g lb; BDE-100, 0.131 ng/g). This suggests that the outliers identified in the Umbria, Trentino, and Piedmont regions (Table 3) constituted isolated cases within their respective regions.

## 4. Conclusions

This study describes the largest dataset of levels of exposure to PBDEs of women of reproductive age in Italy. The BDE-47, BDE-99, BDE-100, and BDE-153 congeners were the most frequently determined congeners in this study and among the most frequently detected in human sera samples worldwide, with some country-specific differences. The average exposure levels of BDE-99 and BDE-100 detected in the study population were apparently the lowest among the industrialized countries; these low levels could be the result of phasing-out procedures. However, exposure to flame retardants beyond the country-specific bans appears to continue because, with some differences related to chemical structures, they are incorporated into diverse applications such as furniture, electronics, and building insulation. After their restriction, the continued presence of flame-retardant-containing products further complicates the determination of exposure sources because different product types have very different replacement rates (e.g., smartphones at 2−6 years vs. building insulation at 30−50 yrs) [50]. All these sources, for most people, appear as if they cannot be changed because they are linked to home or work environments. This, hypothetically, can be correlated to the differences observed between the seven Italian regions. We detected the highest exposure levels in Sicily and the lowest in the Latium region, although the differences in the BDE-99 and BDE-100 concentration ranges did not exceed one order of magnitude. The difference in PBDE concentrations among regions, together with the presence of relatively high concentrations of some PBDEs in individual participants, provides an indication of the need to continue monitoring human exposure to these substances.

## Figures and Tables

**Table 1:** Descriptive statistics of BDE-*47*, BDE-99, BDE-100, and BDE-153 (ng/g lipid) concentrations in serum samples of the study sample of women.

	N	Minimum	P_25_	Median	Geometric Mean	Mean	P_75_	P_95_	Maximum
BDE-47	318	0.01	0.03	0.245	0.169	0.986	0.67	3.09	42.0
BDE-99	347	0.01	0.05	0.100	0.111	0.753	0.230	0.840	156.7
BDE-100	441	0.01	0.06	0.110	0.112	0.200	0.190	0.540	7.20
BDE-153	467	0.04	0.46	0.670	0.654	0.982	0.950	1.71	3.38

**Table 2:** Adjusted means (ng BDE-99/g lipid) calculated from the predictions of the regression models for “BMI” at fixed values of the covariates. The *p*-values in the fourth column refer to the coefficients of the regressions. A significant value means that there is a difference between the concentration detected at the 16.3–18.4 BMI range (the baseline level) and the one detected at the other BMI ranges.

BMI Range	BDE-99 ng/g Lipid		
	Mean	IC95	*p*
16.3–18.4	0.078	0.046–0.110	—
18.5–24.9	0.117	0.103–0.130	0.027
25.0–29.8	0.117	0.088–0.147	0.075
30.1–37.2	0.105	0.050–0.161	0.399

**Table 3:** Outliers of the data distributions of the four congeners in the study population (determined by the modified Z-score).

Sample ID	BDE-47 (ng/g Lipid)	BDE-99 (ng/g Lipid)	BDE-100 (ng/g Lipid)	BDE-153 (ng/g Lipid)	BMI (kg/m^2^)	Age (Years)	Region	Zone	Status	Height (cm)	Weight (Kg)
462	11.8	3.65	1.52	2.22	22.9	24	Umbria	Rural	single	162	60
444	—	—	—	3.38	21.8	24	Trentino	Rural	single	170	63
287	—	—	—	4.17	29.1	37	Umbria	Urban	married	155	70
281	42.0	27.6	3.12	79.8	25.8	30	Umbria	Rural	single	160	66
384	34.9	156	6.23	14.8	20.7	26	Piedmont	Urban	single	163	55
29	31.6	8.87	7.20	9.16	17.7	32	Umbria	Rural	single	168	80

**Table 4:** Adjusted means (ng BDE-99 and BDE-100 ng/g lipid) calculated from the predictions of the regression models for the variable “Region” at fixed values of the covariates. The *p*-values in the fourth and eighth columns refer to the coefficients of the regressions. A significant value means that there is a difference between the concentration detected in Latium (the baseline level) and the one detected in the other regions.

Region	BDE-99 ng/g Lipid			BDE-100 ng/g Lipid		
	Mean	IC_95_	*p*	Mean	IC_95_	*p*
Latium	0.084	−0.051–0.115	—	0.083	0.060–0.105	—
Piedmont	0.099	−0.073–0.118	0.553	0.108	0.092–0.124	0.076
Apulia	0.122	−0.095–0.149	0.072	0.116	0.097–0.135	0.029
Sicily	0.147	0.110–0.184	0.012	0.131	0.105–0.158	0.007
Trentino	0.122	−0.095–0.150	0.069	0.112	0.094–0.130	0.051
Umbria	0.114	−0.090–0.138	0.134	0.119	0.102–0.137	0.013

## Data Availability

The Local Sanitary Units involved in the study retain records of consent in both paper and electronic formats, in compliance with Articles 7 and 23 of Legislative Decree No. 196/03. The ISS retains the database containing the results of the study. Individual data cannot be shared openly to protect study participant privacy.

## Supplementary Materials

The following supporting information can be downloaded at https://www.mdpi.com/article/10.3390/toxics14010072/s1, Table S1. Questionnaire variables considered in the development of the regression analysis with the pertinent missing number.
