# Supplementary material for: Polybrominated Diphenyl Ether (PBDE) Serum Concentrations in Italian Women of Reproductive Age
Source: Toxics. 2026 Jan 13;14(1):72. doi: 10.3390/toxics14010072 (PMC12846057; doi:10.3390/toxics14010072)
Supplement: Supplementary file 1 [file toxics-14-00072-s001.zip › toxics-4021936-supplementary.pdf]

Table S1. Questionnaire variables considered in the development of the regression analysis with the pertinent missing number.

Variable	Missing (n)	Note	Value n
zone	1	—	3
region	1	—	8
civil state	1	—	4
education	2	—	4
address	117	—	356
municipality	91	—	75
heavy traffic (km)	114	distance	3
industry (km)	234	distance	km
waste landfill (km)	230	distance	km
incineration plant (km)	358	distance	km
exposure type	89	—	4
whole milk	63	y/n	—
skimmed milk	53	y/n	—
cheese	49	y/n	—
eggs	17	y/n	—
chiken	10	y/n	—
beef	2	y/n	—
fish	1	y/n	—
crustacean and mollusks	7	y/n	—
animal and veg. fats	9	y/n	—
cooked vegetables	2	y/n	—
raw vegetables	3	y/n	—

Instrumental analysis of PBDE, additional information

MID procedure: PBDE_VF1_PFK

Mid Time Windows:

	Start	Measure	End	Cycletime
# 1	7:00 min	2:00 min	9:00 min	0.35 sec
# 2	9:00 min	1:33 min	10:33 min	0.30 sec
# 3	10:33 min	1:56 min	12:30 min	0.40 sec
# 4	12:30 min	2:00 min	14:30 min	0.40 sec
# 5	14:30 min	2:30 min	17:00 min	0.45 sec
# 6	17:00 min	6:00 min	23:00 min	0.45 sec

Mid Masses:

Window # 1

mass	F	int	gr	time (ms)
242.9851	l	5	1	21
245.9680		2	1	54
247.9661		2	1	54
258.0083		5	1	21
260.0063		5	1	21
268.9819	c	5	1	21
271.8102		5	1	21
273.8072		5	1	21

Window # 2

mass	F	int	gr	time (ms)
480.9691	l	3	1	15
483.7131		1	1	46
485.7111		1	1	46
492.9691	c	3	1	15
495.7533		1	1	46
497.7513		1	1	46

Window # 3

mass	F	int	gr	time (ms)
403.7870		1	1	62
404.9755	l	3	1	20
405.7850		1	1	62
405.8428		2	1	31
407.8398		2	1	31
415.8273		2	1	31
417.8252		2	1	31
430.9723	c	3	1	20

Window # 4

mass	F	int	gr	time (ms)
630.9595	l	3	1	26
641.5320		1	1	80
643.5300		1	1	80
653.5723		2	1	40
654.9595	c	3	1	26
655.5703		2	1	40

Window # 5

mass	F	int	gr	time (ms)
554.9659	l	3	1	24
561.6060		1	1	74
563.6040		1	1	74
573.6462		1	1	74
575.6442		1	1	74
580.9627	c	3	1	24

Window # 6

mass	F	int	gr	time (ms)
792.9499	l	1	1	49
797.3355		1	1	49
799.3335		1	1	49
804.9499	c	1	1	49
809.3757		1	1	49
811.3737		1	1	49
